# Early Diagnosis of Mild Cognitive Impairment Based on Eye Movement Parameters in an Aging Chinese Population

**DOI:** 10.3389/fnagi.2020.00221

**Published:** 2020-07-29

**Authors:** Jing Nie, Qi Qiu, Michael Phillips, Lin Sun, Feng Yan, Xiang Lin, Shifu Xiao, Xia Li

**Affiliations:** ^1^Department of Psychiatry, Shanghai Mental Health Center, Shanghai Jiao Tong University School of Medicine, Shanghai, China; ^2^Departments of Psychiatry and Epidemiology, Columbia University, New York, NY, United States

**Keywords:** mild cognitive impairment, preclinical diagnosis, dementia, eye-tracking assessment, magnetic resonance imaging

## Abstract

**Background**: The pathogenesis of dementia often starts several years prior to clinical onset during which the individual is asymptomatic. Existing strategies for the accurate diagnosis of early dementia are limited by high cost and the invasive nature of the procedures. Eye movement parameters associated with cognitive functions may be helpful in the early identification of dementia and in the development and evaluation of preventive and therapeutic strategies.

**Objective**: We aimed to assess differences in eye movement parameters between healthy elderly individuals and patients with mild cognitive impairment (MCI). Furthermore, we examined the correlations between eye movement parameters with cognitive functions and specific hemispheric region and neural structures in individuals with MCI.

**Method**: Eighty individuals with MCI without dementia (based on DSM-IV criteria) identified by community screening and 170 healthy controls were administered Chinese versions of MoCA and NTB, and a long (20 min) or short (5 min) version of a visual paired comparison (VPC) task. Two weeks later, 44 MCI patients and 107 healthy controls completed a retest of the VPC task, 44 MCI patients and 43 healthy controls among them administered a MRI. At the end of 1-year follow-up, a subset of 26 individuals with MCI and 57 healthy controls were administered the long version of VPC task and MoCA test again. Eye movement parameters and the relationship of eye movement parameters with cognitive functions and with changes in neural structures were compared between groups.

**Results**: Patients with MCI were older, had less education, and had lower scores on cognitive tests than healthy controls. After adjustment for age and level of education, patients with MCI had lower novelty preference scores on the VPC than healthy controls. Using the logistic regression model, the amount of time that subjects focused on these novel images could predict MCI patients from normal elderly with an out of sample area under the receiver operator characteristic curve of 0.62. Furthermore, the cognition score of subjects whose novelty preference score was low decreased more remarkably in 1 year. For both the patient and control groups, VPC novelty preference was significantly correlated with verbal fluency and delayed and short-term memory function. Novelty preference score was also significantly correlated with the cortical thickness of several structures in the right hemisphere.

**Conclusion**: Eye movement parameters are stable indicators to distinguish patients with MCI and cognitively normal subjects and are not affected by different testing versions and numbers. Additionally, the patients’ cognitive deficits and eye movement indices were correlated. Future longitudinal studies should further explore the clinical utility of eye movement parameters as early markers of MCI.

## Introduction

The pathogenesis of dementia is mostly asymptomatic and begins many years before the onset of clinical symptoms. Patients with mild cognitive impairment (MCI) can still perform independent living activities and functions relatively well; however, they have declining verbal memory, visuospatial, and executive capacity (Petersen et al., 1999; Vega and Newhouse, 2014). Previously, MCI was assumed to be an intermediate state between normal aging and dementia that increased the risk of progression to Alzheimer’s disease (AD). However, not all aging processes lead to cognitive impairment. Currently, MCI is regarded as a pathological condition of aging that requires better diagnostic strategies (Petersen, 2004; Galimberti and Scarpini, 2012).

Currently, there are several methods for predicting the progression of MCI, such as structural magnetic resonance imaging (MRI; Wood, 2016), functional imaging techniques (Jessen and Dodel, 2014), and analysis of biomarkers in the cerebrospinal fluid and peripheral blood (Hermida et al., 2012). However, these methods are limited by their high costs and invasive nature; furthermore, they are considered too restrictive for subdiagnosis among the MCI population (Vega and Newhouse, 2014).

The visual paired comparison (VPC) task is a nonverbal recognition task that can sufficiently challenge an individual’s cognitive system, especially the memory. VPC has been shown to have high sensitivity and specificity for distinguishing normal elderly individuals and patients with the MCI. In the VPC task, an item is initially presented briefly, and after a specified time, the subjects are presented with the previously seen item together with a new item. The amount of time spent observing the new item, compared to that spent on the old item, is recorded and the preference for the new item is calculated as the novelty preference (NP) score (Zola et al., 2000; Lagun et al., 2011). Healthy controls tend to concentrate disproportionately more on the novel aspects of the environment (Haque et al., 2019; Jiang et al., 2019; Oyama et al., 2019). In the VPC tasks, the expected normal performance is that the subjects spend more time looking at new pictures than the old ones. In contrast, memory impairment may be characterized by roughly equal amount of time spent reading fiction and familiarizing with pictures, which indicates an impaired declarative memory of previously observed images.

We aimed to establish a baseline pattern of association between cognitive function and eye-tracking parameters in patients with MCI. We primarily aimed to determine whether the NP score could distinguish patients with MCI from cognitively normal elderly individuals. As a secondary objective, we aimed to determine the correlation of eye movement parameters with various specific cognitive regions. Furthermore, we assessed whether eye movement parameters could indicate underlying regional brain dysfunction and help in the identification of underdiagnosed MCI in subsequent follow-up visits.

## Materials and Methods

### Subjects and Clinical Classification

**Figure 1:**
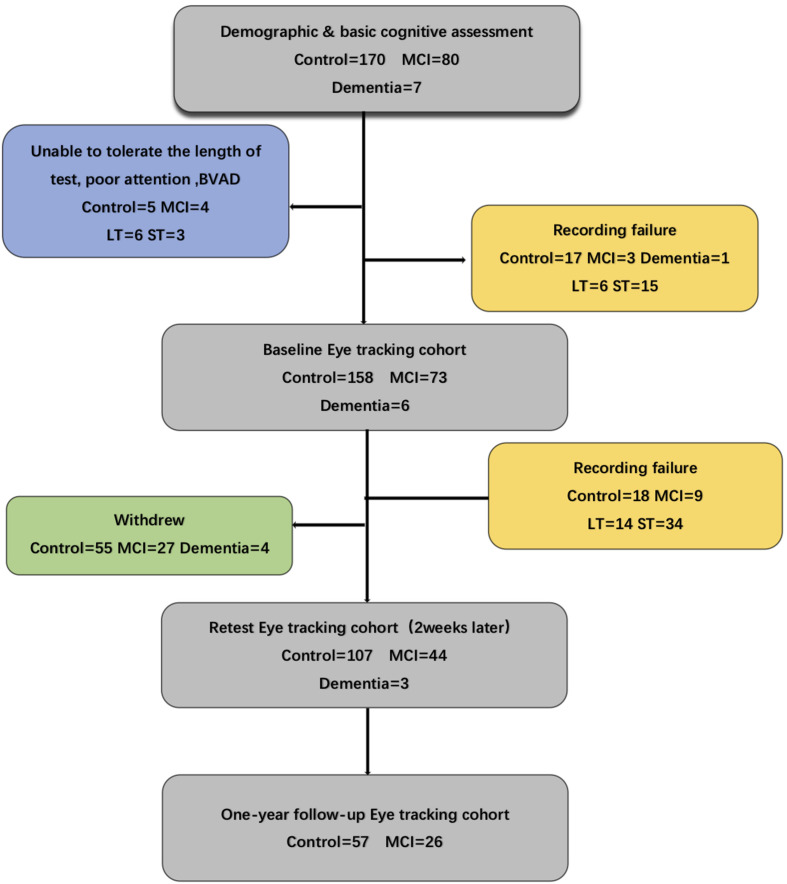
Flow chart for the eye-tracking study indicating recording failure or problems with tolerance to the procedure. Several of the eye-tracking cohorts failed to complete the whole test. MCI, mild cognitive impairment; BVAD, binocular visual acuity differences; LT, long task: eye moving type sets of 30 min; ST, short task: eye moving type sets of 5 min.

In this study, we recruited two groups of participants aged 65–80 years from four different communities in Shanghai and stratified them into MCI (*n* = 80) and healthy (*n* = 170) groups. One-hundred and fourteen participants completed the 12-month follow-up. We excluded subjects with other psychiatric illness; neurological, ophthalmological, or hearing impairment disorders; or inability to sit comfortably because of severe physical illness (Figure 1).

### Diagnostic Procedures

All clinical diagnoses were established by three different geriatric psychiatrists according to the results of standardized assessment and review. Clinical diagnosis of MCI required evidence of definite decline in memory (MoCA scores of >1.5 SD of age-appropriate norms or abnormal memory function for age) and additional possible impairment of other cognitive domains, severity of symptoms or consequent functional limitation not meeting the DSM-IV dementia standard; a diagnosis of NC was made if participants demonstrated no evidence of cognitive decline as compared to their baseline cognitive functions on clinical interview and assessment. Exclusion criteria included a history of substance abuse, learning disability, dementia, and neurological (stroke, tumor, etc.) or psychiatric illness. Moreover, because the VPC task involves visual memory, subjects were excluded if: (1) the eye-tracking equipment could not record adequate pupil and corneal reflection because of physiological constraints or visual problems (such as droopy eyelid, cataracts, detached retinas, glaucoma, extremely small pupils, etc.); and/or (2) participants could not complete the eye-tracking calibration procedure (American Psychiatric Association, 1990). Then, a detailed medical, social, and family history was obtained from each participant; they completed the following subtests: C-MoCA (Chinese Version of Montreal Cognitive Assessment) and C-NTB (Chinese Version of neuropsychological test battery) including WMDS (Wechsler Memory digit span), WMVis-I (Wechsler Memory Scale visual immediate), WMVis-D (Wechsler Memory Scale visual delayed), CFT (Category Fluency Test), COWAT (Controlled Word Association Test), RAVLT-I (Rey Auditory Verbal Learning Test immediate), and RAVLT-D (Rey Auditory Verbal Learning Test delayed). This study was approved by the Institution Review Board of the Shanghai Mental Health Center. Written informed consent was obtained from all the participants or their representatives.

### Assessment of MR Image Acquisition and Processing

MRI scanning was performed using a Siemens Magnetom Verio 3.0T scanner (Siemens, Munich, Germany). We acquired T1-weighted images with 176 sagittal slices using the 3D magnetization-prepared rapid gradient-echo acquisition sequence with the following parameters: TR = 2,300 ms; TE = 2.98 ms; flip angle = 9°; and spatial resolution, 1 mm × 1 mm × 1.2 mm (Lin et al., 2018).

Reconstruction of cortical surfaces and cortical thickness measurements was performed using the automated reconstruction function in the FreeSurfer version 6.0 software as described by Dale et al. (1999).

### Assessment of Visual Exploration

The VPC task requires participants to sit comfortably in front of a monitor and keep their heads positioned on a chinrest to maintain their viewing position. During task performance, all participants’ eye movements were recorded using an Applied Science Laboratories (ASL) Model 5000 remote pan/tilt camera system continuously. A ring of filtered, near-infrared light-emitting diodes illuminated the eye; a high-speed, near-infrared, sensitive charge-coupled device camera captured the pupil and corneal reflection. The gaze angle was determined by the relative positions of the corneal and pupil centers with an accuracy of ±0.75°. The sampling frequency was 60 Hz, with a temporal resolution of 16 ms and linearity of less than 10%. The participants were seated approximately 26 inches from a 19-inch flat panel computer screen that displayed the stimuli. No physical constraints other than a chinrest were used. The eye position was calibrated for each subject using an infrared eye-tracker instrument for 1–2 min. Calibration for each subject was accomplished using a 9-point array. Data of eye fixation and eye movement were recorded with the ASL EYEPOS software. In our present study, we used the dispersion-based fixation detection algorithm from Crutcher MD. The duration threshold was set to 100 ms, and the dispersion threshold was set to five points in eye tracker units (2° of visual angle). The eye fixation and movement data were all recorded with ASL EYEPOS software system. All acquired black and white pictures were high contrast measuring 4.4 inches wide and 6.5 inches high. Unique pictures were used for each trial (Crutcher et al., 2009). System parameters were adjusted until the subject’s fixations were accurately mapped onto the calibration points. Subjects were informed preemptively that images would begin to appear on the computer screen before the test and were instructed to look at the images as if they were watching television. The subjects’ eye movements and fixations were recorded and stored for subsequent analyses. Two types of tasks were used; namely, long and short tasks. The long task (LT) lasted approximately 30 min, whereas the improved short test (ST) required about 10 min, including calibration.

**Figure 2:**
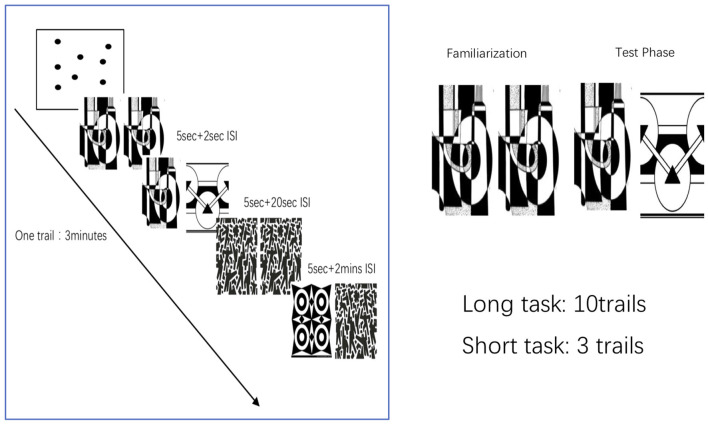
Visual paired comparison task (VPC). Subjects were asked to complete the 9-point calibration procedure before taking the VPC task. During the familiarization phase, participants viewed the two identical pictures presented on the monitor side by side for 5 s. Next, the monitor went dark for a delay interval of either 2 s or 2 min. The test parameters consisted of the presentation of one previously presented image and the novel image. After the test phase, the monitor went dark for 20 s and the next task was initiated. For the long task, participants were administered two blocks of 10 trials (delay order: 2-s delay, 2-min delay) for total 20 trials. The short task contains three trials.

Participants performed a memory paradigm based on eye movements. Each trial in the task consisted of the following two phases: an initial familiarization phase and a subsequent test phase. Subjects were asked to complete the 9-point calibration procedure before taking the VPC task. During the familiarization phase, two identical two-dimensional black-and-white images were presented side by side on the monitor for 5 s. Next, the monitor went dark for a delay interval of either 2 s or 2 min. In the subsequent test phase, the images were again presented side by side for 5 s. One of the images was identical to the previously presented image, whereas the other was a new image. Presentation of the novel image on either the left or right side was randomly selected and equally distributed. After the test phase, the monitor went dark for 20 s and the next task was initiated. To ensure the subject maintained complete attention during the test trials with a 2-min delay interval, the experimenter alerted all the subjects 10 s before the presentation of the next image pair. For the long task, participants were administered two blocks of 10 trials (delay order: 2-s delay, 2-min delay) for a total of 20 trials. The short task contains three trials (Figure 2). The visual search task was used to determine the NP parameter. The eye data parameter called novelty preference (NP) was obtained by calculating the percentage of time spent staring at the novel image by extracting and analyzing the eye fixation and movement data for each participant. The cognitive and eye-tracking assessments were performed on the same day or within a 5-day interval. The whole assessment was completed within approximately 1.5 h, including the rest intervals (Xiao et al., 2013; Pereira et al., 2014).

### Statistical Analysis

Statistical analysis was performed using SPSS Version 22. We used independent sample *t*-tests to perform between-group comparisons of the demographic data, cognitive tests scores. Non-parametric test was applied to compare the rate of decline of MoCA score. The eye-tracking parameters among groups were analyzed by one-way ANOVA followed by the Tukey–Kramer test and multiple comparison adjusted for age and years of formal education. Partial correlation was used to determine the correlation between the novel preference score and the cognitive scores, and cortical thickness. The diagnostic performances of the eye-tracking-based cognitive assessment were determined using a ROC analysis. Statistical significance was set at *p* < 0.05 on a two-tailed test.

## Results

### Demographic and Clinical Characteristics

The mean age of the control group was 71.1 years with a female-to-male ratio of 2.7:1; the mean age of the MCI group was 73.0 years (*p* < 0.001) with a female-to-male ratio of 3.4:1 (*p* > 0.05). The control group had a significantly higher mean c-MoCA scores (25.8) than the MCI group (20.9; *p* < 0.001), and mean NTB scores (218.4) than the MCI group (184.8; *p* < 0.001). All participants with MCI scores (11.8) had lower years of education than the control group (12.8; *p* < 0.001). Moreover, the MCI group performed significantly worse than the normal elderly group in cognitive function tests, especially in verbal fluency (*p* < 0.05), attention (*p* < 0.01), abstraction (*p* < 0.001), and verbal memory domains (*p* < 0.001) in the NTB and C-MoCA tests (Table 1).

**Table 1:** Between-group comparisons of age, years of education, and cognitive scores using independent sample *t* test and comparison of patient sex using Pearson’s *χ*^2^ ± Fisher^’^s exact test.

	Control group (*n* = 170)	MCI group (*n* = 80)	Statistics	*p*-value
Clinical demographic data
Mean (SD) age	71.1 (4.1)	73.0 (4.4)	*T* = −3.4	0.00*
Means (SD) years of education	12.8 (3.1)	11.8 (3.5)	*T* = 2.1	0.04*
Gender (female/male)	131/49	62/18	*χ*^2^ = 0.13	0.42*
Global Cognitive scales			
Means (SD) C-MoCA score	25.8 (2.5)	20.9 (3.2)	*T* = 12.1	0.00*
Means (SD) NTB score	218.4 (27.5)	184.8 (34.5)	*T* = 7.9	0.00*
Specific Cognitive tests—executive function			
Verbal fluency—vegetable	14.5 (3.4)	12.9 (3.8)	*T* = 3.3	0.01*
Verbal fluency—word	9.2 (3.5)	7.1 (2.6)	*T* = 5.5	0.00*
Specific Cognitive tests—Verbal Memory			
Delay recall	11.2 (2.9)	8.0 (3.8)	*T* = 6.5	0.00*
Short-term memory	10.3 (2.4)	8.1 (2.2)	*T* = 6.8	0.00*
Specific Cognitive tests—Visual spatial function	4.5 (0.8)	3.5 (1.2)	*T* = 7.1	0.00*
Specific Cognitive tests—attention	5.7 (0.5)	5.1 (0.9)	*T* = 4.9	0.00*

MCI, mild cognitive impairment; MoCA, *Montreal* Cognitive Assessment; SD, standard deviation. Data are expressed as mean ± SD. *p < 0.05.

### Eye-Tracking Performance

At baseline, the MCI group [0.62 (0.08)] performed significantly worse than the control group [0.66 (0.09)] in the time spent looking at the novel image in the long task (*p* < 0.05 for MCI vs. controls). There was no significant between-group difference in the novelty preference score in the short task [0.57 (0.05) vs. 0.56 (0.07), *p* > 0.05 for MCI vs. controls]. Two weeks later 44 MCI patients and 107 healthy controls completed a retest of the VPC task; in the retest task, compared with cognitively normal elderly individuals, the MCI group had significantly shorter novelty exploration durations in the short task [0.54 (0.05) vs. 0.61 (0.09), *p* < 0.05 for MCI vs. controls; Table 2]. Receiver operating characteristic (ROC) curve analysis revealed an area under the ROC curve (AUC) for novelty preference scores in the long task to be 0.62 (asymptotic significance = 0.03; 95% CI = 0.52–0.73). A cutoff value of 0.605 (a phonemic advantage) indicated 70% accuracy, 72% specificity, 53% sensitivity, 40% positive predictive values (PPV), and 82% negative predictive values (NPV) for the diagnosis of MCI.

**Table 2:** Comparison of mean (SD) novelty preferences scores for long task (20 min) and short task (5 min) between patient and controls groups at baseline and at 2-week follow-up using the *F*-test.

Eye-tracking variables	Control group		MCI group		Statistic*	*p*-value
	*n*	Mean (SD)	*n*	Mean (SD)		
Baseline						
Long task	85	0.66 (0.09)	38	0.62 (0.08)	*F* = 3.34	0.013
Short task	73	0.56 (0.07)	35	0.57 (0.05)	*F* = 0.37	0.829
2-week follow-up						
Long task	51	0.66 (0.10)	23	0.63 (0.08)	*F* = 1.21	0.316
Short task	56	0.61 (0.09)	21	0.54 (0.05)	*F* = 4.47	0.003

*F test is adjusted for age and years of formal education. LT, long task; ST, short task; SD, standard deviation. Data are expressed as mean ± SD, *p < 0.05.

Results of independent sample *t*-test indicated that, based on the three test pairs of long task twice, short task twice, or one long task and short task each, there were no significant between-group differences in the NP score in the repeated long tasks [normal group: 0.65 (0.02) vs. 0.66 (0.02), *t* = 0.3, df = 51, *p* = 0.75; MCI group: 0.62 (0.02) vs. 0.63 (0.03), *t* = 0.2, df = 20, *p* = 0.81] and the repeated short tasks [normal group: 0.58 (0.02) vs. 0.63 (0.02), *t* = 1.9, df = 44, *p* = 0.05; MCI group: 0.55 (0.01) vs. 0.53 (0.01), *t* = 0.68, df = 18, *p* = 0.51; Table 3].

**Table 3:** Comparison of mean (SD) novelty preferences scores for long repeated and short repeated tasks between patient and controls groups at baseline and at 2-week follow-up using the *T*-test.

Eye-tracking variables	Baseline	Two-weeks follow-up	Statistic*	*p*-value
Long repeated task				
HC (*n* = 39)	0.65 (0.02)	0.65 (0.02)	*T* = −0.11	0.915
MCI (*n* = 8)	0.62 (0.02)	0.63 (0.03)	*T* = 1.38	0.217
Short repeated task				
HC (*n* = 30)	0.61 (0.06)	0.62 (0.07)	*T* = −1.60	0.123
MCI (*n* = 10)	0.55 (0.05)	0.56 (0.06)	*T* = −0.74	0.479

HC, healthy control; MCI, mild cognitive impairment; SD, standard deviation. Data are expressed as mean ± SD, *p < 0.05.

### Correlations Between Eye-Tracking Parameters, Cortical Thickness, and Cognitive Functions

**Figure 3:**
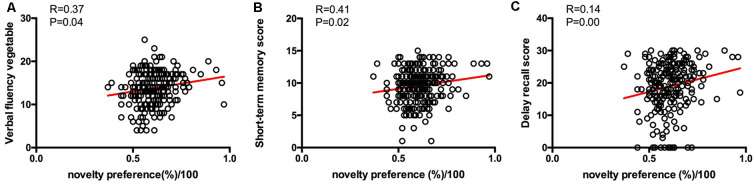
Correlations between eye-tracking parameters and cognitive scores. Only significant findings are shown. **(A)** The novelty preference scores assessed by eye tracking system showed a positive correlation with the verbal fluency score. **(B)** The novelty preference scores assessed by eye tracking system showed a positive correlation with the short-term memory score. **(C)** The novelty preference scores assessed by eye tracking system showed a positive correlation with the delay recall score.

**Figure 4:**
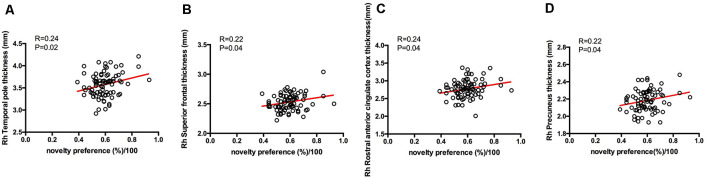
Correlations between eye-tracking parameters and cortical thickness. Only significant findings are shown. Rh, right hemisphere. **(A)** The novelty preference scores assessed by eye tracking system showed a positive correlation with the cortical thickness in temporal pole on the right hemisphere. **(B)** The novelty preference scores assessed by eye tracking system showed a positive correlation with the cortical thickness in superior frontal lobe on the right hemisphere. **(C)** The novelty preference scores assessed by eye tracking system showed a positive correlation with the cortical thickness in rostral anterior cingulate region on the right hemisphere. **(D)** The novelty preference scores assessed by eye tracking system showed a positive correlation with the cortical thickness in precuneus on the right hemisphere.

From the results of partial correlation analysis (with age and years of education as the control factors), novelty preference score correlated positively with performance in the verbal fluency test in the vegetable categories (*R* = 0.37, *p* = 0.04), short-term memory (*R* = 0.41, *p* = 0.02), and delay recall scores (*R* = 0.14, *p* = 0.00; Figure 3). There are no significant differences in the following cortical thickness between the two groups. The MRI results demonstrated a positive correlation between the novelty preference score and cortical thickness in various cerebral regions on the right hemisphere [temporal thickness, superior frontal lobe, rostral anterior cingulate cortex (rACC), and precuneus thickness; *R* = 0.24, *p* = 0.02; *R* = 0.22, *p* = 0.04; *R* = 0.24, *p* = 0.04; and *R* = 0.22, *p* = 0.04; respectively]. The statistically significant correlations are summarized in Figure 4.

### Longitudinal Case–Control Analysis

**Figure 5:**
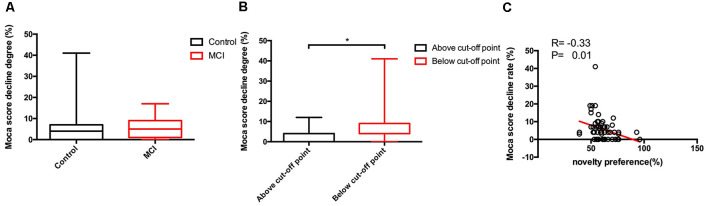
Between-group comparisons of cognitive scores decline degree of different group and the correlations between eye-tracking parameters and cognitive scores decline degree. Only significant findings are shown. Control, normal elderly; MCI, mild cognitive impairment; MoCA, Montreal Cognitive Assessment. Data are expressed as mean ± SD. **(A)** No significant differences of the decline degree of Moca scores in 1 year follow-up were found between control and MCI group. **(B)** Significant differences of the decline degree of Moca scores in 1 year follow-up were apparent between above cut-off point and below cut-off point group. **(C)** The novelty preference scores assessed by eye tracking system showed a negative correlation with the decline degree of Moca scores in 1 year follow-up. *p < 0.05.

One-hundred and fourteen participants completed the 12-month follow-up, 31 of whom completed only the MoCA test, and the rest have completed both the MoCA and the long version of the VPC task. Moreover, nine participants progressed from normal to MCI during the study period, 57 of the subjects are stable normal, and 17 participants are stable MCI (Table 4). There was no difference in novelty preference scores between progressors and non-progressors including stable MCI and normal. Among all the participants who completed the 12-month follow-up, 62 participants show a decline in cognition scores—46 normal elderly individuals and 16 patients with MCI. The degree of decline of MoCA score (%) was calculated as follows: MoCA total score baseline − MoCA total score endpoint)/MoCA total score baseline*100. Results of the non-parametric test showed that there were no significant differences between the MCI and control groups for the degree of decline of cognition [normal group vs. MCI group, 0.04 (0.00–0.07) vs. 0.05 (0.01–0.09), *z* = −1.1, *p* = 0.26; Figure 5A]. Based on the ROC analysis above, we find that the novelty preference scores of 0.605 in the long task had high specificity to discriminate MCI from normal. According to that, 62 participants who took the long task at baseline were categorized into the following two groups: above cutoff point group (*n* = 23) and below cutoff point group (*n* = 39). Cognitive score in the below cutoff point group decreased more than that in the above cutoff point group [above cutoff point group vs. below cutoff point group: 0.04 (0.00–0.04) vs. 0.04 (0.04–0.09), *z* = −2.5, *p* = 0.01; Figure 5B].

**Table 4:** Eye movement indices according to the Group (non-Progressor groups: MCI stable and control stable group, Progressor group) using the Kruskal–Wallis test followed by Steel–Dwass multiple comparison tests.

Eye-tracking variables	Non-progressor Control group		Non-progressor MCI group		Progressor group		*p*-value
	*n*	Mean (SD)	*n*	Mean (SD)	*n*	Mean (SD)	
Novelty preference, in percentages (SD)	57	0.60 (0.08)	17	0.58 (0.08)	9	0.60 (0.10)	0.885
*P*-value		0.903^a^		0.655^b^		0.687^c^

*^a^Non-Progressor Control vs. progressor, ^b^Non-Progressor Control vs. non-progressor MCI, ^c^non-progressor MCI vs. progressor. SD, standard deviation*.

On partial correlation analysis (with age and years of education as the control factor), novelty preference score correlated negatively with the degree of decline of MoCA score (*R* = −0.33, *p* = 0.01; Figure 5C).

## Discussion

In this study, we assessed the NP score in the eye-tracking task as a simple, novel, and non-invasive diagnostic biomarker of MCI. We found that this NP score accurately distinguished patients with MCI from cognitively normal subjects. In our subsequent retest study, we found that eye movement parameters were stable indicators of cognition and were not affected by the paired testing types and testing times. The improved version could reduce data loss and ensure retention of the sensitivity and specificity of the test. Furthermore, participants with poor novelty preference score showed more decline in cognition in 1-year follow-up. Our study suggests that the VPC task in combination with near-infrared eye-tracking might be helpful to explore the underlying dysfunctions in brain regions.

Therefore, although further longitudinal clinical studies are needed, our findings suggest that novelty preference scores in the VPC task could be an easily accessible physiological marker for diagnosis of MCI and might help to identify “seemingly healthy” subjects who are underdiagnosed for MCI.

### Eye Movement Parameters in the MCI Group

Patients with MCI show equally distributed observation times between the new and familiar pictures; however, normal individuals tend to focus disproportionately more on the novel pictures (Berlyne, 1960; Loftus and Mackworth, 1978; Fernández et al., 2018). The original long task allows participants to understand the operational process; however, the short task cannot. Therefore, our major finding from the eye-tracking task was the novelty preference score of the long task, which was low in the MCI group, similar to that reported by Crutcher et al. (2009). It should be noted that diagnosing MCI is more difficult than diagnosing dementia owing to the lack of objective indicators and boundaries between MCI and normal elderly are difficult to ascertain. Our method achieved a specificity of 72% for distinguishing subjects with MCI from normal controls. The results indicated that the VPC task could distinguish patients with cognitive impairment from normal elderly individuals based on the eye-tracking data and that the novelty preference score could probably be used as an objective indicator. Our test can also be suitably expanded to communities for its convenience. However, there remains a need for further improvement of eye movement parameters for the PPV exhibited by our method is a little lower than the values reported for some test for AD subjects (Lagun et al., 2011). In the retest task, the subjects, especially the cognitive-normal elderly individuals, could not concentrate on the test, as observed in the findings from our subjects. Given the increased potential for loss of data because of the poor attention spans and physical condition of elderly individuals, the discriminatory effect was not found in the retest task. Interestingly, patients with MCI who were subjected to a 2-week delay had low novelty preference scores in the short task.

Based on the results of our repeated tasks, eye movement parameters were identified as stable indicators of cognition that remain unaffected by learning effects. Therefore, use of the long task followed by the short task and performing comparisons between the results of the two tasks could sufficiently increase task sensitivity and decrease the physical and psychological burden on subjects. Development of an improved version might further facilitate validity and decrease the failure rate.

Based on the 1-year follow-up results, participants who progressed to MCI during the study period did not show significantly low novelty preference scores. Compared with cognitive-normal elderly individuals, patients with MCI also did not show a larger magnitude of decrease in cognition. Based on the ROC analysis above, we find that the novelty preference scores of 0.605 in the long task has a high specificity for discriminating MCI from normal. According to it, the cognition score of participants, regardless of MCI or normal aging group whose novelty preference score was below the cutoff point, decreased more remarkably in 1 year. It is possible that some healthy control patients were actually undiagnosed cases of MCI. They might have cognition impairment that is not detected by the available scales. This shows that the diagnosis of MCI urgently needs to be combined with objective indicators in the future.

### Correlations Between Cognitive Function, Neuroanatomical, and Eye Movement Parameters

The novelty preference score positively correlated with verbal fluency and verbal memory in both the short-term memory and delay recall tasks. At the neuroanatomical level, the aforementioned cognitive functions are controlled by the temporal and parietal regions of the brain. In our study, 80 of our subjects underwent MRI scanning at baseline, which showed no evidence of acute territorial infarction, mass effect, hemorrhage, infection, or midline shift. We found that the novelty preference score tends to correlate positively with the cortical thickness of specific functional regions of the right brain, including the temporal pole region, superior frontal lobe, rACC, and precuneus. Previously, patients with MCI were reported to have poor performance for episodic memory and executive function, which correlated with the level of atrophy in the right superior temporal pole structures (Ruiz López et al., 2008). Compared to the left hemisphere, the correlations between verb and letter fluency were more robust in the right hemisphere. The temporal lobe is considered the center of verbal recognition memory; furthermore, semantic verbal fluency task is more sensitive to assess temporal region impairments (Henry and Crawford, 2004; Clark et al., 2014; Wong et al., 2018). Previous findings indicate that the VPC task is specifically more sensitive to assess medial temporal lobe (MTL) dysfunction in patients with MCI (Clark et al., 2014). More importantly, a study on patients with schizophrenia indicated decreased cortical thickness in the superior frontal region, which is related to decreased cognitive function and role control (Ong et al., 2005). The rACC plays a key role in the regulation of both emotional and motivational information, as well as in the regulation of emotional response (Tully et al., 2014). A previous study indicated that individuals with late-onset depression and cognitive impairment have decreased cortical thickness of the rACC (Bush et al., 2000). The precuneus not only is involved in self-consciousness but also plays an essential role in the integration of visual–spatial information and retrieval of episodic memory (Lim et al., 2012). The function of the precuneus might be influenced by cortical thinning, early amyloid deposition, and hypometabolic alterations in this region that are induced by the pathologic processes in MCI (Bailly et al., 2015a,b; Haussmann et al., 2017). Taken together, our results show that these regions tend to be closely related to cognitive function and emotional regulation, and reduced cortical thickness may impede their function. Findings of MRI and VPC task provide some evidences of impaired performance in the VPC task preceding detectable structural changes in the memory and emotional control system; however, the correlations between VPC score and MRI findings are weak; additional evidences are needed to support and validate our findings.

### Possible Clinical Practice of VPC Task in Profiling and Predicting Preclinical Dementia

Two to five years before the onset of AD, patients experience slight neurological deficits in cognitive abilities, indicative of MCI. A previous study (Coutinho et al., 2015) reported that damage in the MTL memory system can predict the risk of dementia and that there were related deficits in visual short-term memory (VSTM) between the AD and MCI stage. Given that we observed possible correlation among novelty preference score with temporal–parietal cognitive functions and typical regional thickness, the potential utility of the novelty preference score as an early marker of MCI cannot be ruled out. However, future longitudinal studies are required to confirm these correlations and the ability to identify underdiagnosed cases of MCI.

Despite not being widely available, the measure derived from the device-based VPC task is valuable clinically. As a physiological marker, its performance is not influenced by subjects’ educational and intelligence levels. Our improved eye-tracking tasks were well-tolerated because of their shorter testing times compared to those of the original type. For example, our visual search task required only 5–10 min to complete, including calibration. However, this device has shortcomings in that participants with eyelid apraxia or ophthalmological disorders may have difficulty completing the task.

### Limitations and Future Research Directions

The present study has several limitations. Since there was lack of information on the premorbid intelligence of the participants, the assessed cognitive function may not completely reflect the true cognitive decline. Moreover, it is undeniable that the correlation between NP scores and cognitive tests scores and MRI findings is weak, and more evidences between them are needed in future. MCI patients are quite heterogeneous; however, we did not subdivide different MCI subtypes, like amnestic plus additional cognitive domains. Older-old people (≥75 years old) have an even high risk of dementia, and the mean age of our subjects was 71 years, which was relatively young. We will pay more attention to investigate the characteristics of various types of MCI and expand age range in further research.

In our study, we identified a simple, novel, non-invasive diagnostic biomarker of MCI and established an optimal model to distinguish patients with MCI from cognitively normal elderly individuals in China using a relative large scale. Furthermore, we observed that eye movement parameters may predict the rate of cognition decline. It is worthy to conduct further follow-up assessments of our current subjects to test the robustness of the correlations with disease progression. Since we have already started the follow-up visits and have accumulated the results for 1-year follow up, we hope to further establish the relationship between cognitive functions and eye-tracking parameters and adopt the VPC task as a possible adjunctive measurement to identify underdiagnosed cases of MCI with further follow-up visits.

## Data Availability Statement

All relevant raw data are freely available to any researchers who wish to use them for non-commercial purposes while preserving any necessary confidentiality and anonymity. The datasets are available on request to the corresponding author.

## Ethics Statement

This study was carried out in accordance with the “Shanghai Mental Health Center ethical standards committee on human experimentation” with written informed consent from all subjects. All participants gave written informed consent in accordance with the Declaration of Helsinki. The protocol was reviewed and approved by the “Shanghai Mental Health Center ethical standards committee.” All subjects also gave written informed consent for the publication of this case report.

## Author Contributions

JN and QQ performed statistical analysis and drafted the main manuscript text. LS, XLin, and FY performed the experiments and acquired the data. XLi and SX were involved in study conception, participated in design and coordination, and helped to draft the manuscript. MP was responsible for data acquisition. All authors helped to draft the manuscript and gave critical comments. All the authors are acknowledged.

## Conflict of Interest

The authors declare that the research was conducted in the absence of any commercial or financial relationships that could be construed as a potential conflict of interest.

## References

[B1] American Psychiatric Association (1990). Diagnostic and Statistical Manual of Mental Disorders, Fourth Edition. Washington, DC: American Psychiatric Association.

[B2] Bailly M. Destrieux C. Hommet C. Mondon K. Cottier J. P. Beaufils E.. (2015a). Precuneus and cingulate cortex atrophy and hypometabolism in patients with Alzheimer’s disease and mild cognitive impairment: MRI and (18)F-FDG PET quantitative analysis using FreeSurfer. Biomed Res. Int. 2015:583931. 10.1155/2015/583931 26346648 PMC4539420

[B3] Bailly M. Ribeiro M. J. Vercouillie J. Hommet C. Gissot V. Camus V.. (2015b). 18F-FDG and 18F-florbetapir PET in clinical practice: regional analysis in mild cognitive impairment and Alzheimer disease. Clin. Nucl. Med. 40, e111–e116. 10.1097/rlu.0000000000000666 25549345

[B4] Berlyne D. E. (1960). Conflict, Arousal and Curiosity. New York, NY: McGraw-Hill.

[B5] Bush G. Luu P. Posner M. I. (2000). Cognitive and emotional influences in anterior cingulate cortex. Trends Cogn. Sci. 4, 215–222. 10.1016/s1364-6613(00)01483-2 10827444

[B6] Clark D. G. Wadley V. G. Kapur P. DeRamus T. P. Singletary B. Nicholas A. P.. (2014). Lexical factors and cerebral regions influencing verbal fluency performance in MCI. Neuropsychologia 54, 98–111. 10.1016/j.neuropsychologia.2013.12.010 24384308

[B7] Coutinho A. M. Porto F. H. Duran F. L. Prando S. Ono C. R. Feitosa E. A.. (2015). Brain metabolism and cerebrospinal fluid biomarkers profile of non-amnestic mild cognitive impairment in comparison to amnestic mild cognitive impairment and normal older subjects. Alzheimers Res. Ther. 7:58. 10.1186/s13195-015-0143-0 26373380 PMC4572657

[B8] Crutcher M. D. Calhoun-Haney R. Manzanares C. M. Lah J. J. Levey A. I. Zola S. M. (2009). Eye tracking during a visual paired comparison task as a predictor of early dementia. Am. J. Alzheimers Dis. Other Demen. 24, 258–266. 10.1177/1533317509332093 19246573 PMC2701976

[B9] Dale A. M. Fischl B. Sereno M. I. (1999). Cortical surface-based analysis. I. Segmentation and surface reconstruction. Neuroimage 9, 179–194. 10.1006/nimg.1998.0395 9931268

[B10] Fernández G. Orozco D. Agamennoni O. Schumacher M. Sañudo S. Biondi J.. (2018). Visual processing during short-term memory binding in mild Alzheimer’s disease. J. Alzheimers Dis. 63, 185–194. 10.3233/jad-170728 29614644

[B11] Galimberti D. Scarpini E. (2012). Progress in Alzheimer’s disease. J. Neurol. 259, 201–211. 10.1007/s00415-011-6145-3 21706152

[B12] Haque R. U. Manzanares C. M. Brown L. N. Pongos A. L. Lah J. J. Clifford G. D.. (2019). VisMET: a passive, efficient and sensitive assessment of visuospatial memory in healthy aging, mild cognitive impairment and Alzheimer’s disease. Learn. Mem. 26, 93–100. 10.1101/lm.048124.118 30770466 PMC6380203

[B13] Haussmann R. Werner A. Gruschwitz A. Osterrath A. Lange J. Donix K. L.. (2017). Precuneus structure changes in amnestic mild cognitive impairment. Am. J. Alzheimers Dis. Other Demen. 32, 22–26. 10.1177/1533317516678087 28100076 PMC10852559

[B14] Henry J. D. Crawford J. R. (2004). A meta-analytic review of verbal fluency performance following focal cortical lesions. Neuropsychology 18, 284–295. 10.1037/0894-4105.18.2.284 15099151

[B15] Hermida A. P. Mcdonald W. M. Steenland K. Levey A. (2012). The association between late-life depression, mild cognitive impairment and dementia: is inflammation the missing link? Expert Rev. Neurother. 2012, 1339–1350. 10.1586/ern.12.127 23234395 PMC4404497

[B16] Jessen F. Dodel R. (2014). Prädiktion der Alzheimer-Demenz [Prediction of Alzheimer’s dementia]. Nervenarzt. 85, 1233–1237. 10.1007/s00115-014-4064-0 25231823

[B17] Jiang J. Yan Z. Sheng C. Wang M. Guan Q. Yu Z.. (2019). A novel detection tool for mild cognitive impairment patients based on eye movement and electroencephalogram. J. Alzheimers Dis. 72, 389–399. 10.3233/jad-190628 31594231

[B18] Lagun D. Manzanares C. Zola S. M. Buffalo E. A. Agichtein E. (2011). Detecting cognitive impairment by eye movement analysis using automatic classification algorithms. J. Neurosci. Methods 201, 196–203. 10.1016/j.jneumeth.2011.06.027 21801750 PMC3403832

[B19] Lim H. K. Jung W. S. Ahn K. J. Won W. Y. Hahn C. Lee S. Y.. (2012). Regional cortical thickness and subcortical volume changes are associated with cognitive impairments in the drug-naive patients with late-onset depression. Neuropsychopharmacology 37, 838–849. 10.1038/npp.2011.264 22048467 PMC3260976

[B20] Lin S. Hua X. Jie Z. Wei L. Jing N. Qi Q.. (2018). Alcohol consumption and subclinical findings on cognitive function, biochemical indexes and cortical anatomy in cognitively normal aging Han Chinese population. Front. Aging Neurosci. 10:182. 10.3389/fnagi.2018.00182 29970998 PMC6018200

[B21] Loftus G. R. Mackworth N. H. (1978). Cognitive determinants of fixation location during picture viewing. J. Exp. Psychol. Hum. Percept. Perform. 4, 565–572. 10.1037/0096-1523.4.4.565 722248

[B22] Ong J. C. Seel R. T. Carne W. F. Brown R. Pegg P. O. Jehle P. J. (2005). A brief neuropsychological protocol for assessing patients with Parkinson’s disease. NeuroRehabilitation 20, 191–203. 10.3233/nre-2005-20306 16340100

[B23] Oyama A. Takeda S. Ito Y. Nakajima T. Takami Y. Takeya Y.. (2019). Novel method for rapid assessment of cognitive impairment using high-performance eye-tracking technology. Sci. Rep. 9:12932. 10.1038/s41598-019-49275-x 31506486 PMC6736938

[B24] Pereira M. L. Camargo M. V. Aprahamian I. Forlenza O. V. (2014). Eye movement analysis and cognitive processing: detecting indicators of conversion to Alzheimer’s disease. Neuropsychiatr Dis. Treat. 10, 1273–1285. 10.2147/ndt.s55371 25031536 PMC4096446

[B26] Petersen R. C. (2004). Mild cognitive impairment as a diagnostic entity. J. Intern. Med. 256, 183–194. 10.1111/j.1365-2796.2004.01388.x 15324362

[B25] Petersen R. C. Smith G. E. Waring S. C. Ivnik R. J. Tangalos E. G. Kokmen E. (1999). Mild cognitive impairment: clinical characterization and outcome. Arch. Neurol. 56, 303–308. 10.1037/e314192004-003 10190820

[B27] Ruiz López E. C. Fernández-Garcıá Y. Alemán-Gómez Y. Bobes-León M. A. (2008). Mild cognitive impairment: MRI study combined with cognitive measurements. Clin. Neurophysiol. 119, e125–e126. 10.1016/j.clinph.2008.04.123

[B28] Tully L. M. Lincoln S. H. Liyanage-Don N. Hooker C. I. (2014). Impaired cognitive control mediates the relationship between cortical thickness of the superior frontal gyrus and role functioning in schizophrenia. Schizophr. Res. 152, 358–364. 10.1016/j.schres.2013.12.005 24388000

[B29] Vega J. N. Newhouse P. A. (2014). Mild cognitive impairment: diagnosis, longitudinal course and emerging treatments. Curr. Psychiatry Rep. 16:490. 10.1007/s11920-014-0490-8 25160795 PMC4169219

[B30] Wong W. H. Chan A. Wong A. Lau C. K. Yeung J. H. Mok V. C.. (2018). Eye movement parameters and cognitive functions in Parkinson’s disease patients without dementia. Parkinsonism Relat. Disord. 52, 43–48. 10.1016/j.parkreldis.2018.03.013 29571955

[B31] Wood Heather. (2016). Alzheimer disease: meta-analysis finds high reversion rate from MCI to normal cognition. Nat. Rev. Neurol. 12:189. 10.1038/nrneurol.2016.29 26965671

[B32] Xiao S. Li J. Tang M. Chen W. Bao F. Wang H.. (2013). Methodology of China’s national study on the evaluation, early recognition and treatment of psychological problems in the elderly: China Longitudinal Aging Study (CLAS). Shanghai Arch. Psychiatry 25, 91–98. 10.3969/j.issn.1002-0829.2013.02.005 24991140 PMC4054537

[B33] Zola S. M. Squire L. R. Teng E. Stefanacci L. Buffalo E. A. Clark R. E. (2000). Impaired recognition memory in monkeys after damage limited to the hippocampal region. J. Neurosci. 20, 451–463. 10.1523/JNEUROSCI.20-01-00451.2000 10627621 PMC6774137

